# Antihistamines for Allergic Rhinitis Treatment from the Viewpoint of Nonsedative Properties

**DOI:** 10.3390/ijms20010213

**Published:** 2019-01-08

**Authors:** Hideyuki Kawauchi, Kazuhiko Yanai, De-Yun Wang, Koju Itahashi, Kimihiro Okubo

**Affiliations:** 1 Department of Otorhinolaryngology, Shimane University, Faculty of Medicine, 89-1 Enya-cho, Izumo-shi, Shimane 693-8501, Japan; kawauchi@med.shimane-u.ac.jp; 2 Department of Pharmacology, Tohoku University School of Medicine, 2-1 Seiryo-cho, Aoba-Ku, Sendai 980-8575, Japan; 3 Cyclotron and Radioisotope Center, Tohoku University, 6-3 Aoba, Aramaki, Aoba-ku, Sendai 980-8578, Japan; 4 Department of Otolaryngology, Yong Loo Lin School of Medicine, National University of Singapore, 1E Kent Ridge Road, Singapore 119228, Singapore; entwdy@nus.edu.sg; 5 Medical Science Section, Pharmaceutical R&D Division, Meiji Seika Pharma Co., Ltd., Kyobashi 2-4-16, Chuo-Ku, Tokyo 104-8002, Japan; kouju.itahashi@meiji.com; 6 Department of Otorhinolaryngology, Nippon Medical School, 1-1-5 Sendagi, Bunkyo-ku, Tokyo 113-8602, Japan; ent-kimi@nms.ac.jp

**Keywords:** allergic rhinitis, antihistamine, bilastine, fexofenadine, H_1_ receptor occupancy, non-brain-penetrating

## Abstract

Antihistamines targeting the histamine H_1_ receptor play an important role in improving and maintaining the quality of life of patients with allergic rhinitis. For more effective and safer use of second-generation drugs, which are recommended by various guidelines, a classification based on their detailed characteristics is necessary. Antihistamines for first-line therapy should not have central depressant/sedative activities. Sedative properties (drowsiness and impaired performance) are associated with the inhibition of central histamine neurons. Brain H_1_ receptor occupancy (H_1_RO) is a useful index shown to be correlated with indices based on clinical findings. Antihistamines are classified into non-sedating (<20%), less-sedating (20–50%), and sedating (≥50%) groups based on H_1_RO. Among the non-sedating group, fexofenadine and bilastine are classified into “non-brain-penetrating antihistamines” based on the H_1_RO. These two drugs have many common chemical properties. However, bilastine has more potent binding affinity to the H_1_ receptor, and its action tends to last longer. In well-controlled studies using objective indices, bilastine does not affect psychomotor or driving performance even at twice the usual dose (20 mg). Upon selecting antihistamines for allergic rhinitis, various situations should be taken into our consideration. This review summarizes that the non-brain-penetrating antihistamines should be chosen for the first-line therapy of mild allergic rhinitis.

## 1. Introduction

Allergic rhinitis is one of the type I allergic diseases and IgE-mediated inflammation developing through exposure of the nasal mucosa to allergens [1,2]. When specific IgE antibodies bound to the Fc ε receptors of mast cells or basophils once recognizes an allergen (antigen), histamine, leukotriene, platelet-activating factor, etc. are released from those cells, inducing immediate-type allergic reactions [3,4]. The nasal triad symptoms are paroxysmal and repetitive sneezing, running nose, and stuffy nose [1,2]. Allergic rhinitis is classified into continuous or intermittent types according to the duration of symptoms, and perennial or seasonal (pollinosis) types according to the difference in allergens [1,2]. The estimated number of patients with allergic rhinitis is estimated to be more than 500 million worldwide and 150 million in the Asia-Pacific region [1]. Particularly in Japan, the incidence of allergic rhinitis increased in number from 29.8% in 1998 to 39.4% in 2008, and interestingly, the proportion of pollinosis increased during that period [2].

The treatments for allergic rhinitis could be divided into removal or avoidance of allergens, pharmaceutical treatment, immunotherapy, and surgical intervention. Among these, pharmaceutical treatment employing antihistamines, leukotriene receptor antagonists, topical steroids, vasoconstrictors, etc. play an important role in improving and maintaining the quality of life. In particular, antihistamines (oral, eye drop, and nasal drop formulations) are widely indicated for mild to severe conditions [1,2,5]. In fact, an observational study in Asia reported that patients with allergic rhinitis were most frequently (≥50%) treated with antihistamines (followed by nasal spray steroids; approximately 30%) [6].

Antihistamines have a long history in their development. Antihistamines released earlier in the market are called first-generation antihistamines. Second-generation antihistamines were introduced around 1980 and later [7]. First-generation antihistamines inevitably exhibited central depressant/sedative activities. Moreover, they showed low specificity and adverse reactions (thirst, urinary retention, tachycardia, etc.) due to effects such as anticholinergic effects. Therefore, to overcome these drawbacks, various types of pharmacological improvements were performed during development of second-generation antihistamines [7,8,9].

Currently revised guidelines for the treatment of allergic rhinitis recommend the use of second-generation antihistamines [1,2]. However, because of a larger number of drug products in so called “antihistamines” category, understanding the pharmacological characteristics of individual drugs is essential for effective and safer use of antihistamines in clinical practice. Therefore, in this article, we will review pharmacological characteristics of antihistamines, focusing especially on nonsedative properties as an important point of these drugs. At the same time, we will summarize the characteristics of representative second-generation antihistamines and provide expert opinions regarding the favorable selection of antihistamines for the treatment of patients with allergic rhinitis based on international standards.

## 2. Pharmacological Aspects Related to Sedation of Antihistamines

### 2.1. Histamine and Its Receptors

Histamine is produced as a biological amine from L-histidine through the action of histidine decarboxylase. Histamine-producing cells include histamine neurons, gastric enterochromaffin-like cells, mast cells, and basophils [8]. The action of histamine is exhibited through four types of histamine receptors (H_1_, H_2_, H_3_, and H_4_ receptors, all of which are G-protein-coupled receptors (GPCR)) and is related to neurotransmission, smooth muscle contraction, vascular permeability, gastric acid secretion, basophil functions, etc. [9,10]. H_1_ receptors, to which antihistamines for allergy treatment mainly bind, are distributed in various cells, such as central nervous system cells, smooth muscle cells (blood vessels and respiratory system), endothelial cells, chondrocytes, hepatocytes, dendrocytes, monocytes, neutrophils, and lymphocytes [10]. Incidentally, antihistamines targeting H_1_ receptors (H_1_ antihistamines) are not structurally related to histamine; they are inverse agonists binding to receptor sites that are different from those of histamine [11,12]. Precise information on histamine and its receptors can be obtained from the web site of IUPHAR/BPS Guide to Pharmacology (http://www.guidetopharmacology.org/).

The crystal structure of a complex between the H_1_ receptor and doxepin, a first-generation antihistamine, was elucidated in 2011 [13]. The H_1_ receptor has seven transmembrane helices, which is a characteristic common to GPCR, with the N-terminal outside and the C-terminal inside the cell. The configuration of the transmembrane helices of the receptor is similar to that of other GPCR. However, with regard to the between-helices loop structure, the similarity with other amine receptors is low [13]. Most amino acid residues residing at the doxepin binding site of the H_1_ receptor are highly conserved among amine receptors. However, amino acid residues interacting with phosphate ion are proper to the H_1_ receptor. In the docking model, a carboxyl group (negative charge) of second generation antihistamines (olopatadine, acrivastine, levocetirizine, and fexofenadine) is considered to bind with the binding site of the phosphate ion, and high specificity of these drugs toward the H_1_ receptor was suggested [13]. In the docking model, the carboxyl group of bilastine is also predicted to bind with the binding site of the phosphate ion of the H_1_ receptor [8]. Epinastine, desloratadine, loratadine, and rupatadine, which belong to an amino group type, have low specificity to the H_1_ receptor, and bind also with other GPCR.

### 2.2. Sedative Potentials of Antihistamines and Their Classification Based on Brain H_1_ Receptor Occupancy

Sedative properties of H_1_ antihistamines (drowsiness, impaired performance, etc.) are caused by inhibition of the functions of central histamine neurons [7,8,9]. The cell bodies of histamine neurons are localized in the hypothalamic tuberomammillary nucleus, with their nerve fibers being distributed widely from the whole brain to a part of the spinal cord to form a monoaminergic nerve system [8,14,15]. At an arousal state, histamine neurons are strongly excited to release histamine, and the released histamine strongly activates the function of the cerebral cortex either directly via H_1_ and H_2_ receptors or by exciting the acetylcholine neurons and noradrenaline neurons in the brainstem, the acetylcholine neurons in the substantia innominate, and the glutamine neurons in the hypothalamus. The activation of the cerebral cortex function by histamine neurons is closely associated with the maintenance of the arousal state [16], enhanced cognitive functions, and inhibition of appetite [17].

**Figure 1:**
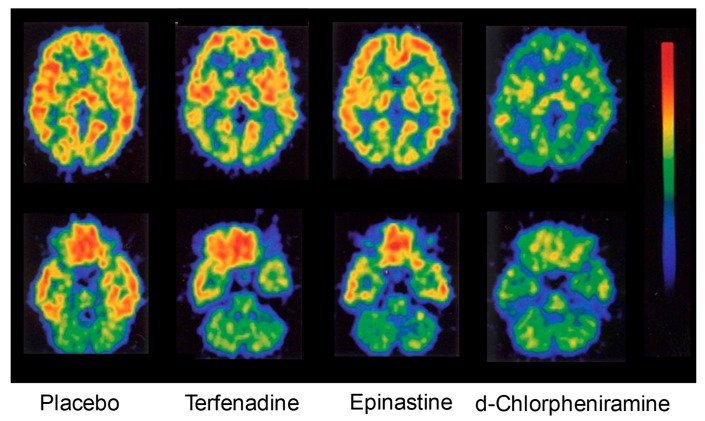
Early studies on positron emission tomography (PET) measurements of brain histamine H_1_ receptor occupancy. These images show radioactivity in horizontal brain sections at the striatal level (upper) and the cerebellar level (lower) after intravenous injection of [^11^C]doxepin into healthy volunteers. Terfenadine (60 mg), epinastine (20 mg), or d-chlorpheniramine (2 mg) was orally administered 1 h before the doxepin injection. For example, d-chlorpheniramine clearly decreased the accumulation of the [^11^C]ligand in the brain, resulting in a histamine H_1_ receptor occupancy of 76.8%. The brain histamine H_1_ receptor occupancy (%) was defined and calculated as described [18]. Modified based on [18].

In order for antihistamines to exhibit sedative properties, they need to penetrate into the brain and bind with H_1_ receptors. Thus, the brain H_1_ receptor occupancy (H_1_RO) has been investigated as an index of sedative potential [18,19,20,21,22]. For the measurement of this index, [^11^C]doxepin and positron emission tomography are used (Figure 1) [18,19,20,21,22,23,24,25]. To cite an example, a study of the second-generation antihistamines fexofenadine and cetirizine [21] reported that the H_1_RO of fexofenadine (120 mg) was minimal (−0.1%), whereas that of cetirizine (20 mg) was moderate (26.0%). In objective psychomotor tests in the same study, fexofenadine did not show significant differences from the placebo, and in some evaluation endpoints, the effects of fexofenadine were significantly less than those of cetirizine. With regard to subjective sleepiness, although cetirizine tended to increase sleepiness, fexofenadine did not show a significant difference from the placebo [21]. On a different note, while the first-generation hydroxyzine (30 mg) significantly prolonged the break reaction time while driving a car compared to the placebo, fexofenadine (120 mg) did not show a significant difference from the placebo [26]. Furthermore, the proportional impairment ratio (PIR) based on subjective feeling and objective performance was investigated as an index of sedation due to H_1_ antihistamines to rank many first- and second-generation antihistamines [27,28]. The results also showed differences in PIR among drugs as follows, fexofenadine, 0.00; cetirizine, 0.25; and hydroxyzine, 2.43 [27]. Thus, correlations among PIR, the incidence rate of sedative effects, and H_1_RO measured by positron emission tomography have been confirmed [8,9].

**Figure 2:**
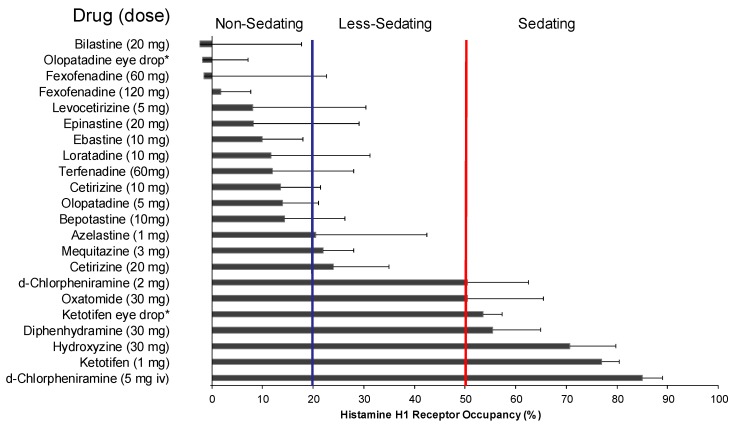
Brain histamine H_1_ receptor occupancies of various antihistamines and classification for sedating actions. The occupancy data are represented as the mean ± SD of measurements in [^11^C]doxepin-positron emission tomography after oral single-dose, eye drop (*), or intravenous (iv) administration of the drugs; the data were obtained by more than one research group. When H_1_ receptor occupancy was 20% or lower, impaired performance was not observed in a simultaneously performed cognitive function test [19,21], and therefore, the drug could be classified as “non-sedating” (Modified based on [8]).

**Figure 3:**
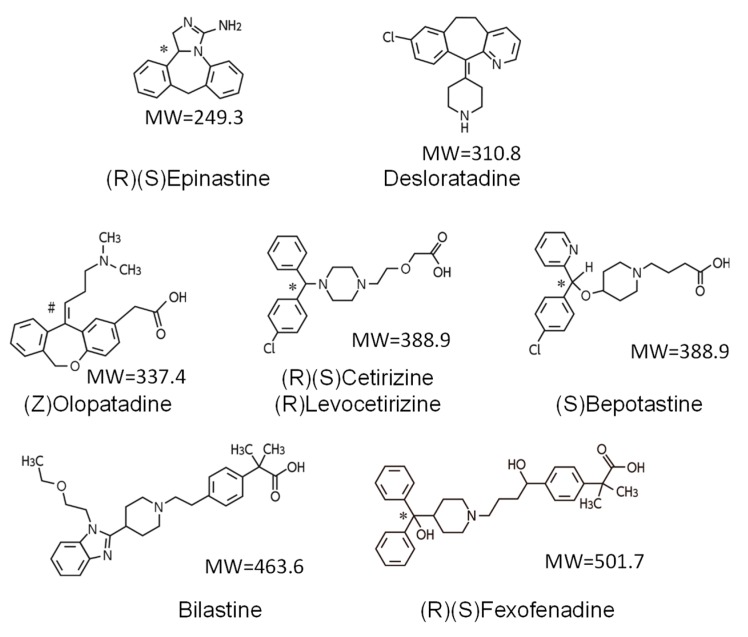
Chemical structures of several non-sedating antihistamines. There are two types of non-sedating antihistamines: the amino group type (epinastine and desloratadine) and the carboxy group type (the others; zwitterionic compounds). MW, molecular weight; (R), (S), optical isomers; (Z), geometric isomer; *, the carbon atom that is related to optical isomerism; #, the double bond that is related to the structurally different geometric isomer (*cis*-*trans* isomer) without optical isomerism. The carboxy group types are characterized by high specificity to H_1_R, while the amino group types bind to other GPCR receptors such as muscarinic receptors. Note that the mean molecular weight of marketed CNS drugs is approximately 310 Da and that the molecular weights of fexofenadine and bilastine are larger than others.

Yanai et al. measured the H_1_RO of many first- and second-generation antihistamines and proposed a classification of these drugs according to the level of the H_1_RO [8,9,29]. The importance of H_1_RO as an index of nonsedative property of antihistamines was also confirmed at the “Consensus Group of New Generation of Antihistamines (CONGA)”, which is an expert meeting sponsored by the British Society for Allergy and Clinical Immunology [30]. Antihistamines are classified into three groups based on the H_1_RO after a single oral administration: non-sedating (<20%), less-sedating (20–50%), and sedating (≥50%) groups. According to the measurement results by multiple research groups, the non-sedating group includes bilastine (20 mg), fexofenadine (60–120 mg), levocetirizine (5 mg), epinastine (20 mg), ebastine (10 mg), loratadine (10 mg), terfenadine (60 mg), cetirizine (10 mg), olopatadine (5 mg), and bepotastine (10 mg) [8] (Figure 2). The chemical structures of H_1_ antihistamines belonging to the non-sedating group are characterized by the presence of hydrophilic functional groups, i.e., carboxyl group (-COOH) and/or amino group (-NH_2_) (Figure 3), which is considered to suppress the penetration through the blood–brain barrier (BBB).

### 2.3. Non-Brain-Penetrating Antihistamines: Bilastine and Fexofenadine

**Figure 4:**
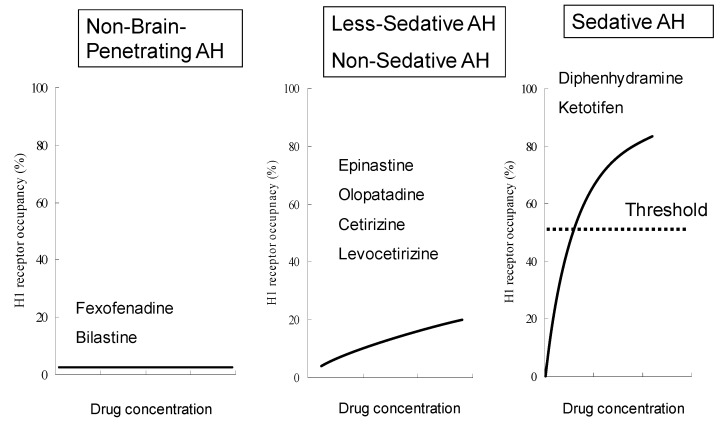
A new classification of antihistamines based on brain histamine H_1_ receptor occupancy. Antihistamines can be classified into non-sedating, less-sedating, and sedating groups based on the H_1_RO and mean plasma concentration of the drugs measured during positron emission tomography, as previously reported [9,29]. In this figure, the concept of “non-brain-penetrating” [32] is included. The H_1_ROs of non-brain-penetrating antihistamines are nearly zero and not correlated to the plasma concentrations of the drugs. The H_1_ROs of non-sedating and less-sedating antihistamines, in the range of up to 20% (for non-sedating) or 50% (for less-sedating), are proportional to some degree to the plasma concentrations of the drugs and have increased brain penetration. Sedative antihistamines rapidly penetrate the brain and show 50% or more H_1_RO, associated with increasing plasma drug concentrations. AH, antihistamines. (Modified based on [9].)

**Figure 5:**
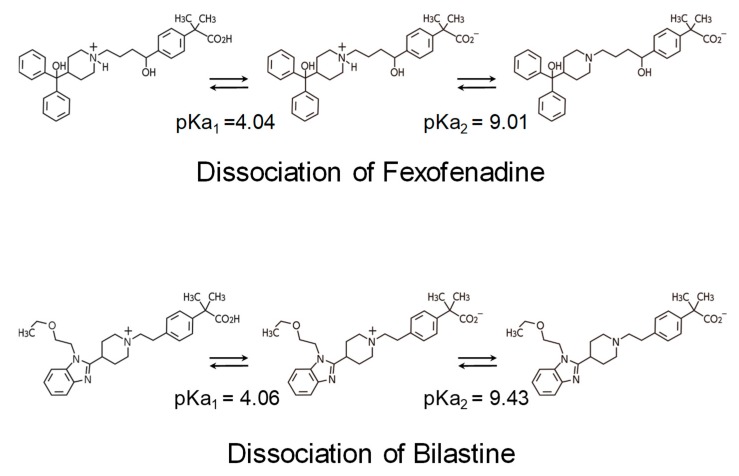
Acid–Base Dissociation Constant (p*K*a). Non-sedating antihistamines of the carboxy group type have zwitterionic properties with positive and negative charges at two sites. The p*K*a of bilastine and fexofenadine are nearly the same, and these drugs are mostly dissociated at the physiological pH, making them difficult to penetrate into the brain. Non-sedating antihistamines are not potent inhibitors of P-glycoprotein; therefore, they are considered not to penetrate into the brain because of complex reasons, including molecular weight and p*K*a.

Among the antihistamines belonging to the non-sedating group, the H_1_ROs of bilastine and fexofenadine, in particular, are nearly 0% [21,22,31] (Figure 2), and these antihistamines minimally penetrate into the brain. Thus, these two drugs can be distinguished as “non-brain-penetrating antihistamines” [32] (Figure 4). Both bilastine and fexofenadine are zwitterions, having both a positive charge (N^+^) and a negative charge (COO^−^) within the molecule. In the docking simulation, their binding modalities with the H_1_ receptor are similar [8]. In addition, the molecular weights of both of these drugs are larger than those of non-sedating antihistamines (Figure 3), their acid–base dissociation constants are similar (bilastine, p*K*a_1_ = 4.06 and p*K*a_2_ = 9.43; fexofenadine, p*K*a_1_ = 4.04 and p*K*a_2_ = 9.01 as predicted values in the DrugBank (https://www.drugbank.ca/)), and they are completely dissociated (ionized) at the physiological pH (Figure 5). Most non-sedating H_1_ antihistamines, including these drugs, are substrates of P-glycoprotein [33,34], and thus their penetration through the BBB is restricted. However, the levels of P-glycoprotein contribution to brain penetration differ depending on individual drugs. In addition to hydrophobicity, molecular weight, and electric charge (net charge under physiological conditions) of the compounds, many other factors, including cytochrome P450 enzymes, enantiomers, etc., are considered to be involved in the BBB or brain penetration [8]. In the case of passive diffusion, brain penetration increases with decreasing molecular weights.

With regard to the binding affinity to the H_1_ receptors, in an in vitro experiment using a Chinese hamster ovary cell line expressing human H_1_ receptors and [^3^H]pyrilamine, the Ki values of fexofenadine, loratadine, cetirizine, olopatadine, levocabastine, and desloratadine were 218, 231, 101, 34, 19, and 3.0 nM, respectively [35]. Although the evaluation system was different, in an in vitro experiment using the inhibition of [^3^H]pyrilamine binding to the H_1_ receptor of a guinea pig cerebellum-derived membrane sample as an index, the Ki values of fexofenadine and bilastine were 246 and 44 nM, respectively [36]. In addition, in an in vitro experiment using HEK293 T cells expressing human H_1_ receptors and [^3^H]pyrilamine, the Ki values of fexofenadine and bilastine were approximately 32 and 8.7 nM, respectively [37]. In summary, among the second-generation antihistamines, while bilastine has a moderate H_1_ receptor affinity, the affinity of fexofenadine is relatively weak; thus, this latter drug needs a higher dosage to exhibit a similar level of activity to bilastine.

### 2.4. Residual Effects by Sedating Antihistamines

The half-life of H_1_ antihistamines in the brain can be longer than that in the plasma; therefore, caution is necessary. Measurement of the H_1_RO 3 to 23 h after the administration of the sedating antihistamines, diphenhydramine (50 mg) and ketotifen (1 mg), suggested that their half-lives were approximately 30 and 45 h, respectively [32]. The half-lives of these drugs in the plasma were 6 to 8 h. Thus, the half-lives in the brain were approximately five times longer than those in the plasma, showing that the half-lives in tissues and those in the blood can be different. It has been reported that sedating antihistamines affect the circadian sleep/wake cycle, delaying the occurrence of REM sleep during sleep or shortening the sleeping time and that drowsiness and impaired performance are observed on the next day as an aftereffect [38]. These observations suggest involvement of brain pharmacokinetics in sedative effects of these drugs. With regard to local administration of sedating antihistamines, it should be well recognized that occupancy of brain H_1_ receptors through eye drop administration has been confirmed [32] (Figure 2) and that brain penetration can also occur through nasal spray [39].

## 3. Clinical Aspects of Non-Sedating Antihistamines

### 3.1. Clinical Profiles of Representative Second-Generation Antihistamines

Clinical profiles of bilastine, fexofenadine, cetirizine, levocetirizine, loratadine, desloratadine, and ebastine are shown in Table 1. Loratadine and desloratadine, belonging to the amino group type, have anticholinergic activity, whereas bilastine, fexofenadine, cetirizine, levocetirizine, and ebastine, belonging to the carboxy group type, show high specificity toward H_1_ receptor antagonistic activity. All of these drugs are indicated for allergic rhinitis and urticaria. These drugs tend to have a short time to maximum plasma concentration (*T*_max_) (within approximately 3 h) and a long elimination half-life (t_½_) (except levocetirizine and loratadine: ≥10 h). The frequency of dosing of fexofenadine is twice daily and that of all other drugs is once daily in Japan. Bilastine, fexofenadine, and cetirizine are not metabolized or are minimally metabolized. Bilastine and fexofenadine do not require dose adjustment according to the level of hepatic dysfunction. These two drugs do not require dose adjustment for patients with renal dysfunction either. As for all the drugs shown in Table 1, caution regarding the induction of drowsiness is required while the patients are being treated. However, they are allowed to drive a car. The antihistamines that most satisfy the requirements for oral H_1_ antihistamines described in the Allergic Rhinitis and its Impact on Asthma (ARIA) guidelines [1] are bilastine and fexofenadine [7]. Among the seven drugs in Table 1, bilastine alone is not indicated for pediatric use (<12 years of age) in Japan. In addition, because bilastine is affected by food ingestion, it is described in the package insert that it should be taken on an empty stomach. Incidentally, it should be noted that non-sedating antihistamines of zwitterion type have the same properties to some degree. Because the organic anion transporting peptides that are associated with the absorption of fexofenadine are inhibited by grapefruit juice [40], concomitant ingestion of the drug and grapefruit juice should be avoided. The absorption of bilastine was similarly decreased by the co-administration of grapefruit juice. Antihistamines should be properly selected for individual patients after fully understanding these characteristics.

### 3.2. Efficacy for Seasonal Allergic Rhinitis

In a multicenter, randomized, double-blind, parallel-group comparative study in 683 patients with seasonal allergic rhinitis (SAR) using oral bilastine (20 mg), cetirizine (10 mg), and placebo once daily for 14 days [41], both bilastine and cetirizine significantly reduced the area under the curve (AUC) of reflective total symptom score (TSS: nasal symptom score [NSS] + non-nasal symptom score [NNSS]) over 14 days of treatment compared to placebo (76.5 and 72.3, respectively, for bilastine and cetirizine, vs. 100.6 for placebo; *p* < 0.001 (Analysis of Variance, ANOVA)). In both drugs, the rates of decreases from the baseline in both NSS (total and individual for nasal obstruction, rhinorrhea, sneezing, and itching) and NNSS (total and individual for ocular tearing, redness, and itching) were nearly the same and were significantly larger than those in the placebo. Furthermore, when the overall discomfort score was used as an index of discomfort associated with allergic rhinitis, the efficacies of bilastine and cetirizine were similar. In this study, the incidence rate of somnolence in the bilastine group was significantly lower than that in the cetirizine group (1.8% vs. 7.5%, *p* < 0.001 [Χ^2^ test]; placebo, 2.2%).

In a multicenter, randomized, double-blind, parallel-group comparative study in 721 patients with SAR using oral bilastine (20 mg), desloratadine (5 mg), and placebo once daily for 14 days [42], bilastine and desloratadine significantly reduced the TSS-AUC compared to placebo (98.4 and 100.5, respectively, for bilastine and desloratadine vs. 118.4 for placebo; *p* < 0.001 [ANOVA]). In addition, when NSS, NNSS, and the score of discomfort associated with rhinitis were used as indices, these drugs showed similar levels of efficacy. In this study, the incidence rates of adverse events in the bilastine group and the desloratadine group were nearly the same.

A randomized, double-blind, 4-way crossover study of bilastine (20 mg), cetirizine (10 mg), fexofenadine (120 mg), and the placebo was conducted in 75 patients with SAR using a Vienna challenge chamber, which enabled artificial exposure to pollens [43]. In this study, drugs were administered 2 h after the start of 6 h-pollen exposure. Significant decreases in the total NSS (TNSS) in individual drug treatment groups compared to the placebo group were observed until 4 h after drug administration. When the patients were again exposed to pollen 22 to 26 h after the drug administration (day 2), the increase in TNSS was significantly inhibited in either drug treatment group compared to the placebo group. The level of the inhibition was stronger in the cetirizine group than in the fexofenadine group. In addition, with regard to the decrease in the amount of nasal secretion and the decrease in the global symptom scale (the composite score for nasal obstruction, rhinorrhea, itchy nose, sneezing, watery eyes, itchy and red eyes, cough, itchy throat, and itchy ears) on day 2, the effects of bilastine and cetirizine were more potent, and their durations of action were longer than those of fexofenadine. Furthermore, in a randomized, double-blind, 4-way crossover study of bilastine (10 or 20 mg), fexofenadine (60 mg, twice with 12 h interval), and placebo in 136 Japanese patients with SAR (Japanese cedar pollinosis) using an OHIO chamber [44], the TNSS of the bilastine 20-mg group was significantly lower than that of the fexofenadine group until 3 h after the treatment.

The efficacy of bilastine in patients with SAR was nearly the same as that of cetirizine and desloratadine and was better than that of fexofenadine [41,42,43,44], and the induction of somnolence by bilastine was weaker than that by cetirizine [41]. Incidentally, although details are not described here, bilastine was shown to be as effective as cetirizine and fexofenadine for perennial allergic rhinitis (PAR) [45,46]. The incidence rates of somnolence due to bilastine (20 mg once daily) and fexofenadine (60 mg twice daily) administered for 2 weeks in Japanese patients with PAR were 0.8% and 0.4% [46].

### 3.3. Central Nervous System Safety of Bilastine

Psychomotor performance was evaluated using multiple objective tests (evaluations of motor activity (Fine Motoric Test, FMT), perception (Critical Flicker-Fusion Frequency Test, CFF), attention (“d2” Cancellation Test, D2T), and associative integration (Simple Reaction Time, SRT)) in a randomized, double-blind, 5-way crossover study in 20 healthy subjects using bilastine (20, 40, or 80 mg), hydroxyzine (25 mg), and placebo once daily for 7 days [47]. Significant psychomotor impairment compared with the placebo was observed after single dose administrations (1 day) of 80 mg bilastine and hydroxyzine. More tests showed significant results with hydroxyzine (SRT, CFF, and D2T) than with bilastine (SRT and CFF). However, after repeated administration (7 days), no significant psychomotor impairment was observed with either of the drugs. Regardless of single or repeated administration, no psychomotor impairment was observed with 20 and 40 mg bilastine.

In a randomized, double-blind, 4-way crossover study in 22 healthy subjects using bilastine (20 or 40 mg), hydroxyzine (50 mg), and placebo once daily for 8 days [48], the effect on driving performance was evaluated using standard deviations of lateral position (SDLP, a measure of car weaving [49]) as an index. Hydroxyzine alone showed significantly larger SDLP compared to placebo both after a single dose (day 1) and repeated dose (day 8). No significant change in SDLP was observed in either 20 or 40 mg bilastine.

In a randomized, double-blind, 6-way crossover study in 24 healthy subjects using concomitant bilastine (20 or 80 mg), cetirizine (10 mg), or hydroxyzine (25 mg) and alcohol (0.8 g/kg), alcohol alone (+drug placebo), and placebos (alcohol placebo and drug placebo) [50], psychomotor performance was evaluated by objective tests (including FMT, CFF, D2T, and SRT). Significant psychomotor impairment compared to the placebo was observed in any of the concomitant administrations. Significant psychomotor impairment compared to alcohol alone was observed in any of the concomitant administrations of 80 mg bilastine, cetirizine, or hydroxyzine with alcohol. The levels of psychomotor impairment with 80 mg bilastine and hydroxyzine were nearly the same, and the level with cetirizine was a little lower than the levels of the above. The level of psychomotor impairment was nearly the same between concomitant bilastine (20 mg) and alcohol and alcohol alone.

As discussed above, bilastine at the usual dose (20 mg) and twice the dose (40 mg) did not show impaired performance due to sedative activity [47,48]; in addition, the drug did not show alcohol interaction at its usual dose (20 mg) [50].

## 4. Conclusions

The H_1_RO is useful as an index of antihistamines, and based on it, antihistamines have been classified into non-sedating, less-sedating, and sedating groups. Among the drugs in the non-sedating group, bilastine and fexofenadine do not show occupancy of the brain H_1_ receptors at their usual doses; thus, they can be referred to as “non-brain-penetrating antihistamines”. In addition to the fact that they are both zwitterions, bilastine and fexofenadine are chemically very similar in many aspects. However, bilastine has a higher affinity to the H_1_ receptor than fexofenadine. Because no substantial differences in clinical efficacy are observed among representative second-generation antihistamines, one of the important points in selecting these drugs is brain penetration and the presence or absence of sedative effect, which are associated with safety. Bilastine has been shown not to affect psychomotor performance and driving performance even at the dose of 40 mg, which is twice the usual dose, by well-controlled studies using objective indices, and thus is considered to be a useful drug for allergic rhinitis. Currently, however, bilastine is not indicated for pediatric use in Japan.

## 5. Expert Opinion

As for antihistamines used for the treatment of allergic rhinitis, drugs not only of “the second generation” but those that have been confirmed to be non-sedating should be recommended [30,51]. The first-generation antihistamines generally have potent sedative effect and also other adverse effects (adverse events such as anticholinergic effect); however, their efficacy is not necessarily more potent than that of second-generation antihistamines. The term “second generation” refers to the classification based on the era they were developed, and thus not all the second-generation antihistamines are non-sedating or less-sedating. It should be noted that sedating drugs, such as ketotifen, can be included in the second-generation drugs (Section 1 and Section 2.4). Incidentally, ingestion of sedating antihistamines as a sleep-inducing drug before sleep should be avoided, because the use of such drugs is likely to deteriorate the quality of sleep and also because the effect may continue to the next day (Section 2.4). The efficacy of second-generation antihistamines against allergic rhinitis is mostly similar (Section 3.2); however, it is important to select the drugs to be used in view of nonsedative properties. Fexofenadine, bilastine, desloratadine, and loratadine are recommended in the “Guidelines for the Handling of the Drugs Used for Aircraft Crew” prepared recently by the Ministry of Land, Infrastructure, and Transport of Japan. However, as for loratadine, a mild sedative effect is reported based on studies of H_1_RO and cognitive functions. Therefore, further study is necessary.

Although the utility of H_1_RO in assessing the sedative potential of antihistamines has been widely recognized, there still remain issues to be addressed further. The reason why H_1_RO is usually measured in healthy young men is because the amount of brain H_1_ receptors (the amount of bound [^11^C]doxepin) is different between sexes and ages. For example, the amount is greater in women than in men, and it decreases with increasing age [8,25]. Details are not known regarding, for example, whether the H_1_ROs in patients with allergic diseases are the same as those in healthy people, or whether the results of repeated administration are correlated with those of conventional single dose administration, despite the observation that, depending on the drug, H_1_RO is increased by repeated administration [52]. However, based on the past findings, the correlations between the H_1_RO at single dose administration of various antihistamines and the evaluation indices of sedation based on clinical findings have been shown. Therefore, the results from the current measurement methods are definitely useful (Section 2.2).

According to the classification based on the H_1_RO, fexofenadine and bilastine can be considered to be distinguished from other second-generation antihistamines as non-brain-penetrating antihistamines. In addition to their chemical properties, these two compounds have similar adaptability to the requirements of ideal antihistamines described in the ARIA guidelines [1]. However, there are some differences between the two drugs: bilastine has a stronger H_1_ receptor binding potency than fexofenadine; the dosing frequency of bilastine is once daily, while that of fexofenadine is twice daily; and bilastine is yet to be indicated for pediatric use (Section 2.3 and Section 3.1). Judging from efficacy results from clinical studies, pharmacological findings regarding non-sedating properties, and clinical study results, bilastine may be one of the best options for H_1_ antihistamines for allergic rhinitis (Section 2.3, Section 3.2, and Section 3.3). The safety and tolerability of 10 mg bilastine once daily for 12 weeks in children (≥2 to <12 years of age) have already been confirmed [53]; thus, its indication expansion to pediatric use is expected in Japan.

With regard to bilastine, impaired performance was not observed even at the dose of 40 mg, which is twice its usual dose, in healthy subjects (Section 3.3). The EAACI/GA^2^LEN/EDF/WAO guidelines regarding urticaria [54] recommend that, for the secondary treatment, the amount of modern second-generation H_1_-antihistamines be increased by four times, citing the evidence from bilastine [54]. However, generally, increasing the dose of bilastine to 80 mg should be avoided as much as possible because, despite its excellent efficacy against urticaria and histamine-induced skin symptoms [7,55,56,57], a study reported that the drug affected psychomotor performance at 80 mg [47]. It will be of significance to obtain the H_1_RO of bilastine when the dose is increased to 40 and 80 mg.

In the ARIA guidelines, the efficacy in nasal congestion, etc. are listed as requirements for the efficacy of oral H_1_ antihistamines [1]. In other words, these drugs are considered to have concomitant anti-inflammatory effects [11,12]. In fact, a basic study using H_1_ receptor gene knockout mice reported that H_1_ receptor blockage inhibits the Th1 cytokines, interferon and IL-2, and increases the Th2 cytokines, IL-4 and IL-13 [58]. However, because clinical effects considered to be due to anti-inflammatory effect are mostly empirical, the selection of antihistamines should be based on the potency of the activity on H_1_ receptors. When efficacy seems inadequate at usual doses, it may be effective to increase the dose. However, risks associated with central depressant/sedative activities, anticholinergic effects, etc. should be thoroughly considered. With regard to the addition of other antihistamines or concomitant use of antiallergic drugs with different mechanisms of action, drug–drug interaction can be a problem. Therefore, H_1_ antihistamines should preferably be devoid of metabolism by the cytochrome P450 system or inhibition of the system [1,30,51]. Bilastine, which is minimally metabolized in the body, satisfies these requirements [7,56].

Communication between physicians and patients is important for the treatment of allergic rhinitis [1,2]. Understanding of not only the symptoms but also the life pattern and degree of treatment satisfaction of the patients and their desire regarding economy/cost may also be necessary. When prescribing H_1_ antihistamines, physicians should confirm concomitantly used drugs if any, select non-sedating antihistamines with as few drug–drug interactions as possible, and explain the benefits and risks of the selected drug to the patients.

### Article Highlights Box

- In selecting antihistamines for allergic rhinitis, it is particularly important for safety that the selected drug does not have central depressant/sedative properties and anticholinergic effects.
- Differences in sedative effects and anticholinergic effects were observed among the second-generation antihistamines.
- Based on the brain H_1_ receptor occupancy, which is an index of sedative properties, fexofenadine and bilastine belonging to the non-sedating group can be distinguished as “non-brain-penetrating antihistamines”.
- No major differences in efficacy are observed among recent, representative, non-sedating antihistamines for allergic rhinitis.
- Central nervous system safety of antihistamines needs to be evaluated not only by subjective indices, such as drowsiness, but also by the results of objective performance tests.
- Non-brain-penetrating antihistamines have been confirmed not to show sedative properties even at twice the usual dose and thus are considered to be the first-line antihistamines for allergic rhinitis.

## Figures and Tables

**Table 1:** Clinical profiles of representative second-generation H_1_ antihistamines *.

Characteristic	Bilastine	Fexofenadine	Cetirizine	Levocetirizine	Loratadine	Desloratadine	Ebastine
H_1_ receptor selectivity	+++	+	+	++	+	++	++
Affinity for H_2/3_ receptors	±	±	±	±	±	±	+
Metabolism	Not metabolized	±	±	++	+++	+++	+++
t_max_ (h)	1.3	1–3	1.0	0.9	1.0–1.5	3.0	2.6–4.0 (carebastine metabolite)
t_1/2_ (h)	14.5	11–15	10.0	7.9	8.4	27.0	15–19 (carebastine metabolite)
Indicated for allergic rhinoconjunctivitis?	Yes	No	Yes/No (some but not all formulations)	No	No	No	No
Indicated for allergic rhinitis?	Yes	Yes	Yes	Yes	Yes	Yes	Yes
Indicated for urticaria?	Yes	Yes	Yes	Yes	Yes	Yes	Yes
Pediatric indication?	No (ongoing studies)	children > 3 years	children 6–12 years	children > 2 years	children > 2 years	children > 1 year	children > 2 years
Dosage adjustment in renal impairment? †	No	No	Yes (in moderate to severe)	Yes (in moderate-to-severe)	Yes	Caution (severe impairment)	Caution
Dosage adjustment in hepatic impairment?	No	No	Yes (if concomitant renal dysfunction)	Yes (if concomitant renal dysfunction)	Yes (severe disease)	Not mentioned	Caution (in mild to moderate)
Dosage adjustment in elderly?	No	No	No (if renal function OK)	Yes (for concomitant moderate-to-severe renal impairment)	No	Not mentioned	No
Interaction with food?	Yes (give on empty stomach)	Not mentioned	No	No	No	No	No
Use in pregnancy and lactation?	Caution (very limited data)	No	Caution	Caution	No	No	No
Clinically relevant drug interactions?	No	Yes (antacids)	No	Unlikely (no available data)	Potential (with inhibitors of CYP3A4 and CYP2D6)	No	Caution
Interaction with alcohol?	No	Not mentioned	Caution	Caution	No	No	No
Can patients drive and operate machinery (i.e., lack of sedative potential)?	Yes (caution: drowsiness)	Yes (impairment unlikely)	Yes (check drug response when intending to drive)	Yes (check drug response when intending to drive)	Yes (caution: drowsiness)	Yes (caution: drowsiness)	Yes (caution: somnolence)
Contraindications	None	None	Severe renal impairment	Severe renal impairment	None	None	Severe hepatic impairment
Number of ARIA recommended antihistamine properties ‡	10	9.5	6	6.5	6.5	6.5	6.5

* This table originates from [7] and is partially modified. Originally, data were obtained from Summary of Product Characteristics for each individual compound (available from http://www.medicines.org.uk/emc/). † Based on the Japanese New Drug Application Review Report and the package insert. ‡ Score is derived from ARIA recommended antihistamine properties [1] (0.5 is given for each characteristic where “caution” is recommended). ±, negligible; +, mild; ++, moderate; +++, marked. t_max_, time to peak plasma concentration; t_1/2_, elimination half-life; ARIA, Allergic Rhinitis and its Impact on Asthma; CYP, cytochrome P450.

## Abbreviations

H_1_RO	Brain H1 receptor occupancy
GPCR	G-protein-coupled receptors
PIR	proportional impairment ratio
CONGA	Consensus Group of New Generation of Antihistamines
BBB	blood–brain barrier
p*K*a	Acid–Base Dissociation Constant
*T*_max_	time to maximum plasma concentration
t_½_	elimination half-life
ARIA	Allergic Rhinitis and its Impact on Asthma
SAR	seasonal allergic rhinitis
TSS	total symptom score
NSS	nasal symptom score
NNSS	non-nasal symptom score
PAR	perennial allergic rhinitis
FMT	Fine Motoric Test
CFF	Critical Flicker-Fusion Frequency Test
D2T	“d2” Cancellation Test
SRT	Simple Reaction Time
SDLP	standard deviations of lateral position
